# Case Report: Lower back pain revealing a hidden gynecologic infection: *Brucella melitensis* superinfection of a giant ovarian cyst—a case report from Palestine

**DOI:** 10.3389/frph.2026.1953959

**Published:** 2026-10-30

**Authors:** Majd Mohsen, Razan Sharaf, Dareen Njoom, Masara Samara, Mohammad Bourini, Saadah S. Jaber, Rabee Adwan

**Affiliations:** 1 Faculty of Medicine, Al-Quds University, Jerusalem, Palestine; 2 Department of Medicine, Faculty of Medicine and Allied Medical Sciences, An-Najah National University, Nablus, Palestine; 3 Department of Internal Medicine, Makassed Hospital, Jerusalem, Palestine; 4 Department of Obstetrics and Gynecology, Makassed Hospital, Jerusalem, Palestine; 5 Department of Infectious Diseases, Makassed Hospital, Jerusalem, Palestine

**Keywords:** brucellosis, giant ovarian cyst, Palestine, serous cystadenoma, superinfection

## Abstract

**Introduction:**

Human brucellosis is a multisystem disease with a broad clinical presentation caused by Brucella species. It commonly presents with nonspecific symptoms that may overlap the manifestations of other diseases. Genitourinary involvement occurs in approximately 10% of cases.

**Case presentation:**

An 18-year-old female presented with severe lower back pain associated with fever, chills, nausea, and night sweats. In addition to constipation, myalgia, arthralgia, and menstrual irregularities. Notably, she reported a history of unpasteurized dairy products one month prior to symptom onset. Abdominal examination revealed right lower quadrant and suprapubic tenderness with a large palpable abdominal mass. Accompanied by thoracolumbar and lumbosacral tenderness.

**Intervention and outcome:**

The patient underwent a left ovarian cystectomy via mini-laparotomy. Histopathological examination revealed a benign serous cystadenoma, while the cultures of blood and cystic fluid were positive for Brucella species, confirming superinfection of the cyst. She demonstrated marked clinical improvement following anti-brucellar treatment and surgical intervention.

**Conclusion:**

This report highlights the importance of considering atypical presentations of a common infectious disease, brucellosis, in endemic regions. It underscores the value of comprehensive clinical assessment and meticulous history-taking in recognizing unusual disease manifestations, thereby facilitating timely diagnosis and reducing the risk of diagnostic delay.

## Introduction

Human brucellosis is a globally distributed zoonotic infection that remains endemic in the Mediterranean region, including Palestine (1). It is caused by *Brucella* species and is transmitted to humans through the consumption of unpasteurized milk and dairy products, ingestion of undercooked meat, or cutaneous inoculation among individuals in direct contact with cattle (2).

Brucellosis is a multisystem disease with a wide clinical presentation. It often presents with nonspecific symptoms such as fever, night sweats, and malaise that are documented in 78% of patients and may overlap with the clinical presentation of other disorders (3). Brucellosis complications may involve any organ system. However, genitourinary involvement is relatively uncommon, occurring in approximately 10% of cases (4).

We report the first documented case from Palestine of a giant ovarian cyst superinfected with *Brucella melitensis* in an 18-year-old female patient presenting with severe low back pain. To the best of our knowledge, this represents the largest *Brucella*-superinfected ovarian cyst reported in the literature and the first described within a benign serous cystadenoma. This atypical presentation expands the recognized spectrum of female genitourinary brucellosis and highlights the importance of considering uncommon infectious etiologies in patients from endemic regions.

## Case presentation

An 18-year-old previously healthy female presented with a 4-day history of severe lower back pain associated with tactile fever, chills, nausea, and reduced oral intake. These symptoms were preceded by one month of drenching night sweats, intermittent chills, myalgia, arthralgia, and constipation. She also reported a missed menstrual cycle during the preceding two months and ingestion of unpasteurized dairy products approximately one month before symptom onset.

On presentation, the patient was in pain but alert, oriented, and hemodynamically stable. Abdominal examination showed right lower quadrant and suprapubic tenderness with a large palpable mass. Musculoskeletal examination demonstrated marked thoracolumbar and lumbosacral tenderness with movement restriction without focal neurological deficits. The remainder of the examination was unremarkable.

Laboratory investigations demonstrated a white blood cell count of 9.4 × 10⁹/L, hemoglobin of 12.2 g/dL, platelet count of 407 × 10⁹/L, ESR of 78 mm/hr, and CRP of 43 mg/L. Renal and liver function tests were within normal limits. CA125 was normal. *Brucella* serological testing was initially performed using the Rose Bengal test as a screening assay, followed by the Standard Agglutination Test (SAT), which demonstrated a high antibody titer of 1:640. Blood cultures showed initial growth after 48 h, and *Brucella* species were identified after 5 days of incubation using the VITEK system. Subsequently, the cultured isolate was sent to the Palestinian National Central Laboratory for PCR confirmation, which identified the species as *B. melitensis*.

Abdominal ultrasound revealed a large fluid-filled cystic lesion without solid components **(**Figure 1**)**.

**Figure 1:**
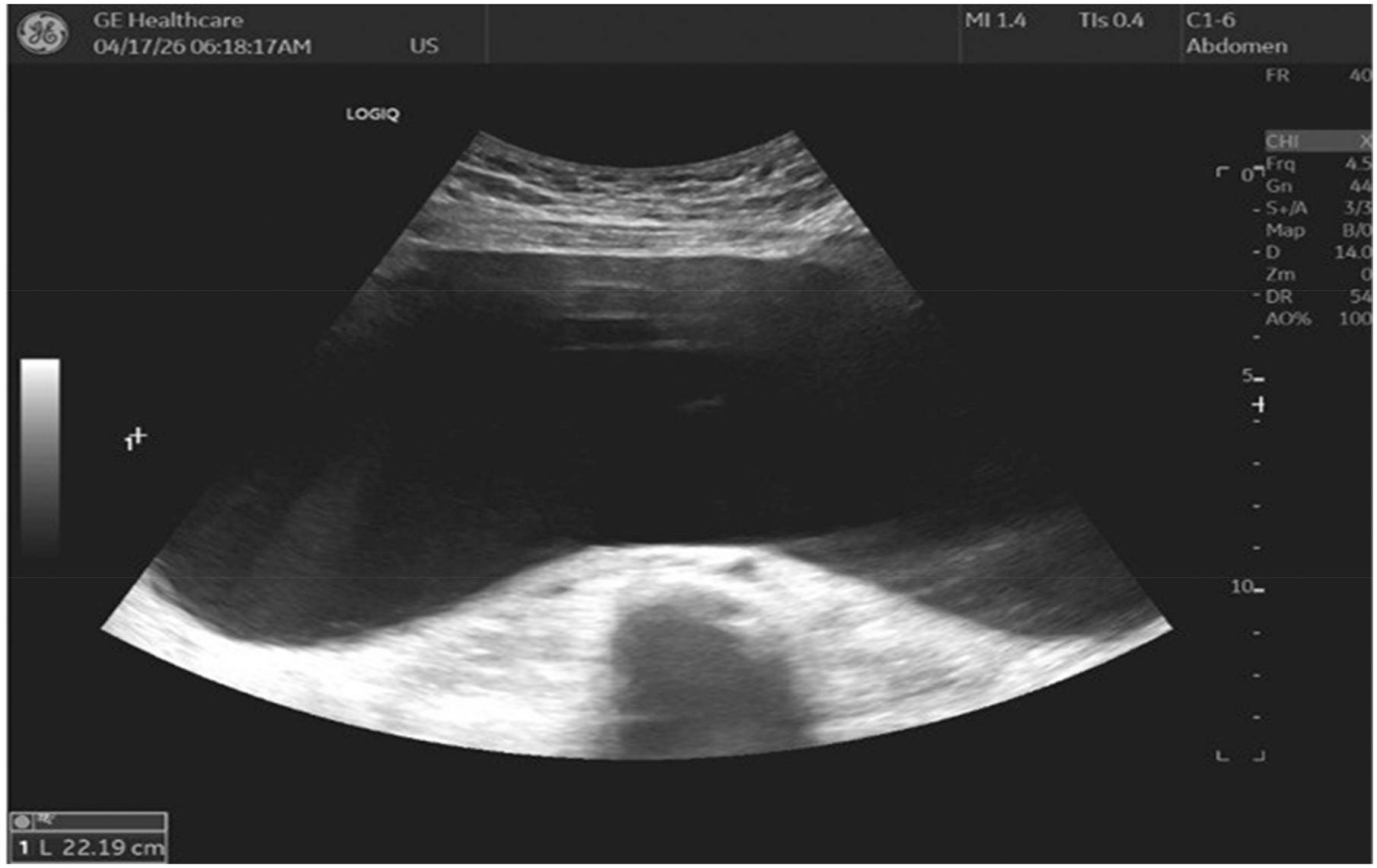
Abdominal ultrasound shows a large, fluid-filled cystic lesion without solid components, extensively occupying the abdominal cavity.

A contrast-enhanced CT scan of the abdomen and pelvis confirmed a large purely cystic mass measuring approximately 27 × 20 × 10 cm, causing bilateral ureteric compression with minimal hydronephrosis. No lymphadenopathy, ascites, or organomegaly were identified **(**Figure 2**)**.

**Figure 2:**
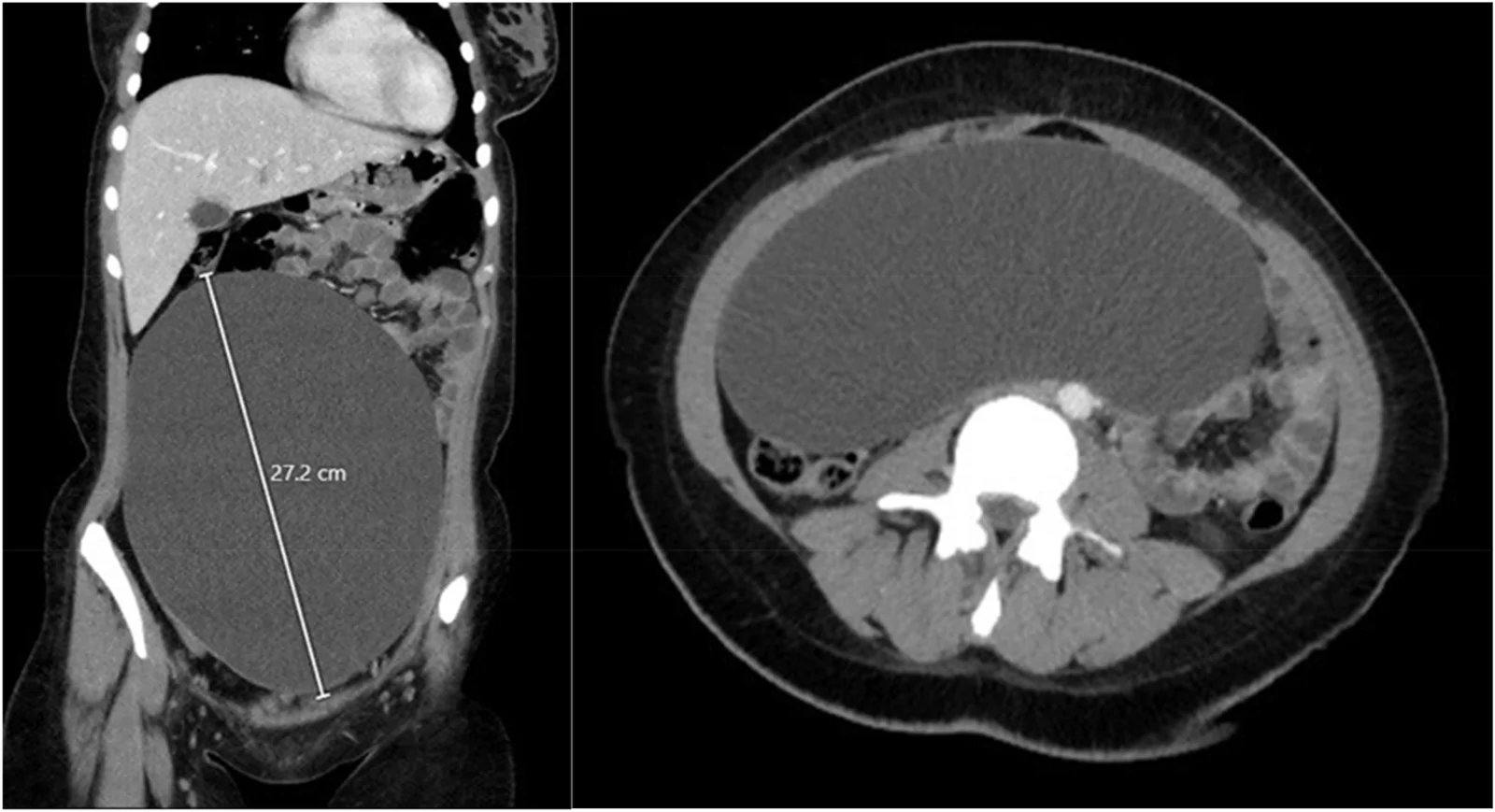
A contrast-enhanced CT scan of the abdomen and pelvis confirmed a large purely cystic mass measuring approximately 27 × 20 × 10 cm.

Given the severe back pain, a whole-spine MRI was performed and showed no evidence of discitis, epidural abscess, spinal cord compression, or other spinal pathology **(**Figure 3**)**.

**Figure 3:**
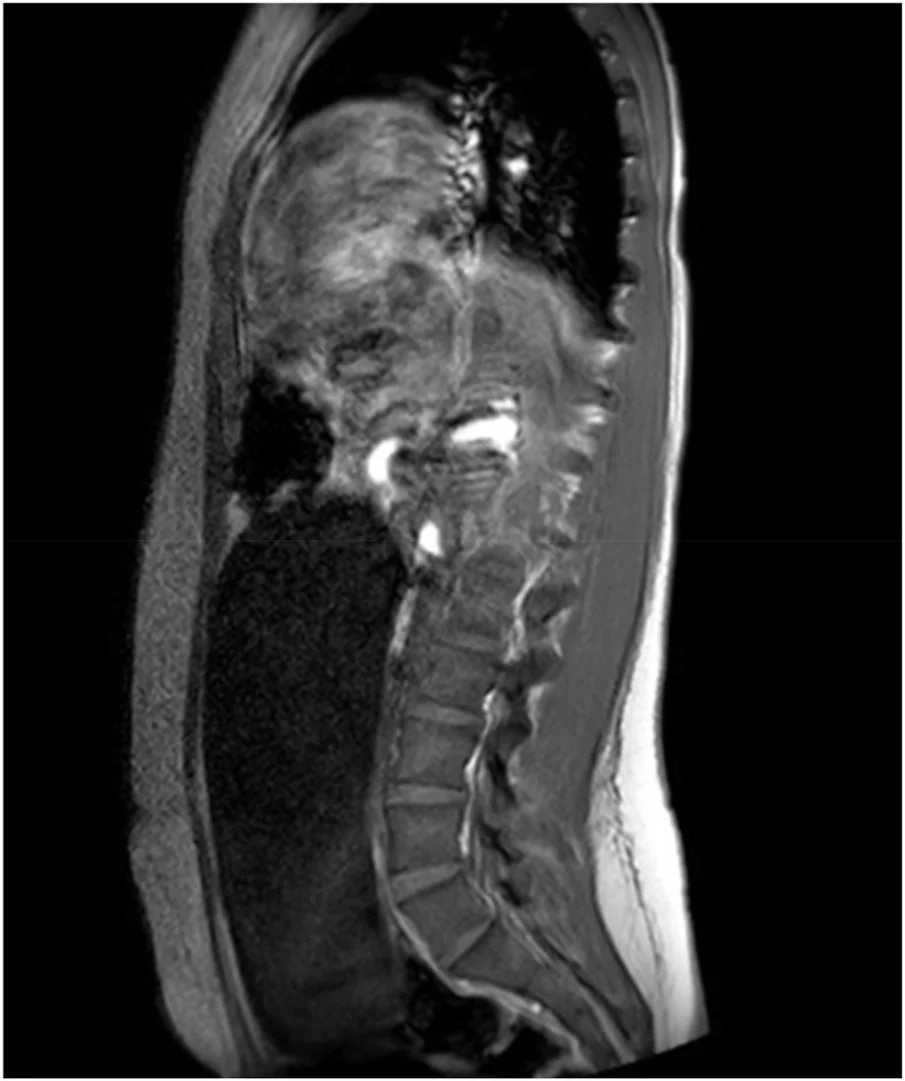
Whole-spine MRI demonstrated no identifiable evidence for discitis, epidural abscess, or spinal cord compression.

Based on the clinical presentation and serological findings, a diagnosis of human brucellosis was established. A triple therapy regimen was initiated with intravenous gentamicin for 10 days combined with oral doxycycline and rifampicin, planned for a total treatment duration of 6–12 weeks based on the patient's clinical status during follow-ups**.**

The patient subsequently underwent left ovarian cystectomy via mini-laparotomy with preservation of normal ovarian tissue. Cyst fluid was aspirated and sent for histopathological and microbiological examination. Histopathological examination demonstrated a benign serous cystadenoma, while cystic fluid culture yielded *Brucella* species, confirming the diagnosis of human brucellosis with superinfection of a giant ovarian cyst.

Postoperatively, the patient showed significant clinical improvement, with substantial relief of her back pain; however, limitations in movement persisted. She was discharged in stable condition on oral rifampicin 600 mg and doxycycline 100 mg for a six-week course. In further follow-ups, she remains clinically well and continues improvement in symptoms and movement limitations.

## Discussion

Brucellosis, also known as Malta fever, is a multisystem disease caused by *B. melitensis, Brucella abortus*, and *Brucella suis,* which are transmitted through ingestion of unpasteurized dairy products or contact with infected animals (3). Recent studies have reported a higher global incidence of brucellosis than previously estimated, with approximately 1.6–2.1 million new human cases annually (5). In Palestine, brucellosis remains a major public health concern. A long-term descriptive epidemiological study conducted by Amro et al. in the West Bank reported 7,935 cases during the study period, with an annual incidence rate per 100,000 population ranging from 17.9 in 2000 to 15.7 in 2020 (6). However, these figures may not fully reflect the actual disease burden, as underreporting may occur due to challenges in healthcare access and disease surveillance, particularly in the setting of ongoing conflict.

Brucellosis has a wide spectrum of clinical manifestations that commonly presents with nonspecific symptoms that are documented in 78% of patients (4). Regarding its ability to disseminate hematogenously, brucellosis may affect any body organ, resulting in diverse and sometimes atypical clinical presentations.

Musculoskeletal involvement represents the most common complications, with reported manifestations including arthralgia (65%), myalgia (56%), and back pain (49%) (4). Sacroiliitis and spondylitis are also well-recognized osteoarticular manifestations, with prevalence ranging from 12% to 36% (4, 7). In contrast, genitourinary involvement is relatively uncommon, occurring in only 10% of patients (4). Epididymo-orchitis is the most common manifestation in males (8), whereas involvement of the female reproductive system is particularly rare (3, 8).

Ovarian involvement in brucellosis is a rare manifestation. Although isolated cases of *Brucella*-associated tubo-ovarian abscesses have been reported (3, 9), only a limited number of cases of *Brucella*-superinfected ovarian cysts have been documented, emphasizing the rarity of this presentation (9–11) (Table 1). Previously reported cases of *Brucella*-superinfected ovarian cysts have predominantly occurred in middle-aged women and involved mature cystic teratomas measuring approximately 10–12 cm. In contrast, our patient was substantially younger and presented with a 27-cm ovarian cyst, representing the largest cyst reported to date. Furthermore, histopathological examination identified a benign serous cystadenoma, which, to the best of our knowledge, represents the first reported case of such an ovarian tumor subtype.

**Table 1:** Summary of reported cases of brucellosis causing ovarian complications.

Profile	Symptoms	Complaining duration	Type of lesion	Size of Cyst	Type of organism	Management	Outcomes
*our case 18 years old	Fever, night sweats, myalgia, arthralgia menstrual abnormality severe low back pain constipation	2 months duration	benign serous cystadenoma	27 × 20 × 10 cm in the left ovary	*Brucella melitensis*	Oral doxycycline 100 mg and rifampicin 600 mg, planned for a total treatment duration of 6–12 weeks based on the patient's status	The patient shows significant improvement and recovery during further follow-ups.
37 years old (10)	Fever and chills	1-Month duration	dermoid cyst	12*11*7 cm in the left ovary	*Brucella melitensis*	After the failure of the initial treatment and the persistence of symptoms, doxycycline 100 mg and rifampicin were started for 3 months.	The patient shows complete recovery and resolution of symptoms.
43 years old (11)	High fever, chills, nausea, vomiting, and right lower quadrant tenderness	Not mentioned	dermoid cyst	12-cm cyst in the right ovary	*Brucella* spp. (unspecified)	Despite receiving suitable antimicrobial agents, symptoms persisted; therefore, treatment was switched to doxycycline and rifampicin for 2 weeks postoperatively and discontinued after 8 weeks.	The patient shows significant improvement and symptom resolution
29 years old (9)	Fever, right-lower quadrant abdominal pain, headache, and vomiting	6 months' duration with a previous history of brucellosis for 2 years	Bilateral tubo-ovarian abscesses and dermoid cyst in the right ovary	A 10 × 10 cm	*Brucella melitensis*	doxycycline and rifampicin. were started and continued for 12 weeks after discharge	Good recovery on follow-up ultrasound with no residual ovarian abscess, and no brucellosis recurrence observed.

The pathogenesis of ovarian cyst superinfection remains poorly understood. In our patient, hematogenous dissemination in the setting of bacteremia represents the most likely mechanism of infection, as supported by positive blood and cystic fluid cultures for the same *Brucella* species.

Moreover, our patient presented primarily with severe low back pain, unlike previously reported cases, in which abdominal or pelvic pain was the main presenting symptom. Given the high frequency of musculoskeletal involvement, spinal brucellosis was initially suspected. However, whole-spine MRI excluded spondylodiscitis and epidural involvement, thereby highlighting the significant mass effect of the giant cyst.

Effective treatment of human brucellosis generally requires combination antibiotic therapy with agents capable of penetrating macrophages and maintaining activity within the acidic intracellular environment. Combination regimens are preferred because monotherapy is associated with a higher risk of relapse (12). Therefore, a limited number of antibiotics are considered effective, including doxycycline, rifampicin, aminoglycosides (gentamicin or streptomycin), fluoroquinolones (levofloxacin or ciprofloxacin), and trimethoprim–sulfamethoxazole (9).

## Conclusion

This report highlights the importance of considering atypical presentations of a common infectious disease, brucellosis, in endemic regions. It underscores the value of comprehensive clinical assessment and meticulous history-taking in recognizing unusual disease manifestations, thereby facilitating timely diagnosis and reducing the risk of diagnostic delay.

## Data Availability

The original contributions presented in the study are included in the article/Supplementary Material, further inquiries can be directed to the corresponding authors.
